# Measles in jails and prisons

**DOI:** 10.1016/S2468-2667(24)00074-4

**Published:** 2024-04-10

**Authors:** Lawrence A Haber, Alysse G Wurcel, Justin Berk

**Affiliations:** Denver Health and Hospital Authority, Denver, CO 80204, USA (LAH); Tufts Medical Center, Boston, MA, USA (AGW); Alpert Medical School at Brown University, Providence, RI, USA (JB)

Approximately 2 million individuals are detained in jails and prisons in the USA, according to Vera statistics. Black people are disproportionately affected by mass incarceration, with nearly a third of Black men facing imprisonment during their lifetime. The medical treatment that individuals receive within the criminal-legal system can either impair or advance health equity. Unfortunately, people who are incarcerated have a high burden of chronic medical illness, a shorter life expectancy, and suboptimal rates of vaccination compared with their counterparts living in the community.^1,2^

With the number of measles cases increasing internationally, attention should be centred on the threat of measles outbreaks in carceral settings. Measles is a highly infectious airborne virus that can lead to severe complications, including pneumonia, encephalitis, and death. Despite the US Centers for Disease Control and Prevention declaring measles eliminated from the USA in 2000, a national resurgence peaked at 1274 cases in 2019, the highest annual number since 1992.^3^ In the first 2 months of 2024 alone, 35 cases of measles have been identified across 15 states.^4^ At the end of 2023, WHO reported that the European region saw a 30-fold increase in measles cases, with increasing related hospitalisations and deaths.^5^ In recent decades, even preceding vaccine hesitancy surrounding the COVID-19 pandemic, rates of measles vaccinations decreased. Models predict that approximately 21% of US children are susceptible to measles, with current levels of protection below herd immunity thresholds.^3^

Continued systems of mass incarceration superimposed on decreasing herd immunity could inevitably fuel measles outbreaks in jails and prisons. Children who were not vaccinated 10–20 years ago are now entering the close congregate quarters of adult carceral facilities. Within cellblocks and communal living spaces, measles can circulate via respiratory droplets and contaminated surfaces, infecting susceptible hosts. Similar risk has been observed within jails, prisons, and immigration detention centres, where overcrowding has led to transmission of viral and mycobacterial infections, such as mumps and tuberculosis.^6^ As witnessed during the first wave of the COVID-19 pandemic, airborne disease can cause morbidity and mortality not only for people who are detained but also for staff and the community surrounding jails and prisons, as individuals cycle through these facilities back into the general population.^7^

We recommend several actions to decrease measles-related diseases and deaths for people held or working in carceral settings. First, we suggest documentation of serological protection against measles as an employment requirement of all people working in jails and prisons. Second, carceral systems should incorporate measles, mumps, and rubella vaccines into the vaccinations offered during intake and clinical examination. Third, public health policy should prioritise efforts to reduce the number of people held in custody and the rate of imprisonment at federal, state, and municipal levels, stemming such reservoirs for infection at the source.
